# Association of Nuclear Factor-Erythroid 2-Related Factor 2, Thioredoxin Interacting Protein, and Heme Oxygenase-1 Gene Polymorphisms with Diabetes and Obesity in Mexican Patients

**DOI:** 10.1155/2016/7367641

**Published:** 2016-05-04

**Authors:** Angélica Saraí Jiménez-Osorio, Susana González-Reyes, Wylly Ramsés García-Niño, Hortensia Moreno-Macías, Martha Eunice Rodríguez-Arellano, Gilberto Vargas-Alarcón, Joaquín Zúñiga, Rodrigo Barquera, José Pedraza-Chaverri

**Affiliations:** ^1^Department of Biology, National Autonomous University of Mexico (UNAM), 04510 Mexico City DF, Mexico; ^2^Economy Department, Autonomous Metropolitan University-Iztapalapa, 09340 Mexico City, DF, Mexico; ^3^Research Department, Regional Hospital “Lic. Adolfo López Mateos”, ISSSTE, 01030 Mexico City, DF, Mexico; ^4^Molecular Biology Department, National Institute of Cardiology “Ignacio Chávez”, 14080 Mexico City, DF, Mexico; ^5^Department of Immunology, National Institute of Respiratory Diseases “Ismael Cosío Villegas”, 14080 Mexico City, DF, Mexico; ^6^Molecular Genetics Laboratory, National School of Anthropology and History, 14030 Mexico City, DF, Mexico

## Abstract

The nuclear factor-erythroid 2- (NF-E2-) related factor 2 (Nrf2) is abated and its ability to reduce oxidative stress is impaired in type 2 diabetes and obesity. Thus, the aim of this study was to explore if polymorphisms in Nrf2 and target genes are associated with diabetes and obesity in Mexican mestizo subjects. The rs1800566 of NAD(P)H:quinone oxidoreductase 1 (NQO1) gene, rs7211 of thioredoxin interacting protein (TXNIP) gene, rs2071749 of heme oxygenase-1 (HMOX1) gene, and the rs6721961 and the rs2364723 from Nrf2 gene were genotyped in 627 diabetic subjects and 1020 controls. The results showed that the rs7211 polymorphism is a protective factor against obesity in nondiabetic subjects (CC + CT versus TT, OR = 0.40, *P* = 0.005) and in women (CC versus CT + TT, OR = 0.7, *P* = 0.016). TT carriers had lower high-density lipoprotein cholesterol levels and lower body mass index. The rs2071749 was positively associated with obesity (AA versus AG + GG, OR = 1.25, *P* = 0.026). Finally, the rs6721961 was negatively associated with diabetes in men (CC versus CA + AA, OR = 0.62, *P* = 0.003). AA carriers showed lower glucose concentrations. No association was found for rs1800566 and rs2364723 polymorphisms. In conclusion, the presence of Nrf2 and related genes polymorphisms are associated with diabetes and obesity in Mexican patients.

## 1. Introduction

The International Diabetes Federation has reported that there are 382 million people living with diabetes worldwide [1]. Type 2 diabetes (T2DM) is a public health problem in developing countries with a direct impact in the economic and social sectors. Being overweight and obesity are risk factors to develop T2DM and could explain the dramatic increase in the incidence and prevalence of T2DM [2, 3]. Both, obesity and T2DM, feature insulin resistance and atherogenic lipid profiles such as increased cholesterol and triglycerides and decreased high-density lipoprotein cholesterol (HDL-C). It is widely accepted that oxidative stress is a common mechanism in the development and progression of these pathologies, with an increased free radical production and reduced antioxidant capacity [4, 5].

The master antioxidant regulator, the nuclear factor-erythroid 2- (NF-E2-) related factor 2 (Nrf2) is a member of the cap'n'collar family of basic leucine zipper transcription factors that regulates the expression of many antioxidant genes including NAD(P)H:quinone oxidoreductase 1 (NQO1) and heme oxygenase-1 (HMOX1) to avoid oxidative damage [6]. Also, it has been demonstrated that Nrf2 activity is abated in diabetes and factors like age, body weight, and blood glucose could modify its activity [7–9], but genetic factors have been poorly studied. Many single nucleotide polymorphisms (SNPs) have been identified in the Nrf2 gene [10]. In particular, the rs6721961 (C-617C) polymorphism has been associated with oxidative stress and risk of newly diagnosed T2DM [7] and increased blood pressure [11]. The rs2364723 (C107G) polymorphism was associated with reduced risk of cardiovascular mortality [12]. The NQO1 gene polymorphism rs1800566 (C609T) is characterized by the replacement of proline by serine at position number 187 of the functional protein, which causes destabilization and inactivation of the enzyme [13, 14]. The rs1800566 polymorphism has been associated with lower levels of blood coagulation factors [15], higher risk of coronary artery disease in T2DM patients [16, 17], and increased triglycerides levels and decreased HDL-C levels in individuals with metabolic syndrome [18]. Polymorphisms in the promoter region of the HMOX1 gene such as rs2071746 (T-413A) and (GT)_*n*_ microsatellite have been associated with various human diseases [19]; however, the rs2071749 polymorphism (A/G) is not associated with hypertension and/or blood pressure in hypertensive patients [20]. It has been reported that the endogenous inhibitor of the thioredoxin (TXN) system, the thioredoxin interacting protein (TXNIP), can be suppressed by Nrf2 [21, 22]. The rs7211 polymorphism in TXNIP was associated with inhibition of TNX, glucose homeostasis, diabetes, and hypertension [23]. Individuals carrying the T allele of the rs7211 polymorphism in the TXNIP gene showed higher plasma triglycerides levels [24].

However, despite the recent efforts to understand the association of antioxidant gene polymorphism with T2DM, the number of studies is still very limited in Mexican population. Thus, the aim of this study was to investigate potential associations between Nrf2 (rs6721961 and rs2364723), NQO1 (rs1800566), HMOX1 (rs2071749), and TXNIP (rs7211) polymorphisms in a T2DM population.

## 2. Materials and Methods

### 2.1. Study Design and Subjects

A case control study was performed which included 1647 Mexican mestizo subjects. This study was approved by the Ethics Committee on Human Studies from the Committee on Research, Ethics and Safety of the Hospital Regional “Lic. Adolfo López Mateos” with registration number 254.2013 and conducted in accordance with the Declaration of Helsinki. Written informed consent was obtained from all subjects who were recruited in Family Medical Clinics of the Instituto de Seguridad y Servicios Sociales de los Trabajadores del Estado (ISSSTE) in Mexico City.

A standard questionnaire was applied to obtain demographic information, family and personal health history (diabetes and/or further diseases), and information about physical activity, alcohol consumption, smoking, and drugs consumption. Their medical history data, weight (kg), and height (m) were obtained. The body mass index (BMI) was estimated by dividing weight by the square of height. Criteria for inclusion/exclusion of participants were as follows: the study included men and women aged 35 and older, nonpregnant and nonlactating women, and subjects without excessive alcohol consumption. The criteria for classification and diagnosis of diabetes were according to the standards in medical care on diabetes from American Diabetes Association (ADA). Subjects of the control group (*n* = 1020) had fasting glucose <5.5 mmol/L and glycated hemoglobin (HbA1c) <5.7%. Subjects with T2DM (*n* = 627) had plasma glucose >7.0 mmol/L and HbA1c >6.5%. Subjects with mental health problems (senile dementia and Alzheimer and Parkinson's disease) and cancer were also excluded. The recruitment period was from May 2012 to October 2014. A total of 1627 individuals were recruited that self-reported Mexican mestizo ancestry (three generations). To each individual the information was given about the protocol and those who decided to enter gave written informed consent to participate. Anthropometric data (waist circumference, height, and weight) and arterial pressure measurements were obtained before blood collection.

### 2.2. Blood Collection and Biochemical Analyses

 Whole blood samples (20 mL) were collected from patients and controls (with fasting of 8–12 h) in order to determine the levels of glucose, triglycerides, total cholesterol, HDL-C, low-density lipoprotein cholesterol (LDL-C), and creatinine in serum using an automatized analyzer (Miura 200, ISE, Rome, Italy). Moreover, total blood with ethylenediaminetetraacetic (EDTA) acid as anticoagulant was used to obtain genomic DNA.

### 2.3. Genotyping

Genomic DNA from whole blood containing EDTA was isolated by standard techniques [25]. The rs2364723 (Nrf2), rs1800566 (NQO1), rs7211 (TXNIP), and rs2071749 (HMOX1) SNPs were genotyped using predesigned 5′ exonuclease TaqMan genotyping assays on a 7500 series Real-Time PCR system, according to manufacturer's instructions (Applied Biosystems, Foster City, CA, USA). The rs6721961 (Nrf2) TaqMan probe was designed according to manufacturer's directions.

### 2.4. Short Tandem Repeats (STRs) Genotyping and Admixture Estimations

Fifteen autosomal STR markers (CSF1PO, FGA, THO1, TPOX, VWA, D3S11358, D5S818, D7S820, D8S1179, D13S317, D16S539, D18S51, D21S11, D19S433, and D2S1338) along with amelogenin were genotyped in 200 controls and 200 cases, using the AmpFlSTR Identifiler Kit (Applied Biosystems, Foster City, CA, USA) as previously described [26].

We performed admixture estimations using the STR's allele distribution by a model-based clustering method with the* Structure* software v. 2.3.4, assuming *k* = 3 populations and 1 × 10^4^ dememorisation steps. The estimations of Amerindian, European, and African components in our Mexican studied groups were performed using the distribution of STRs alleles in different populations including Spaniards [27], Fang Africans [28], and a Native American pool of Huastecos [29] and Tepehuas [30] from the central region of Mexico, who were considered as parental populations.

### 2.5. Statistical Analyses

Continuous variables were analyzed using a *t*-test and were presented as mean ± standard deviation (SD). For categorical variables, a Chi-square test or Fischer exact test was applied and data was presented as percentage.

The Hardy Weinberg equilibrium was calculated in controls using StatCalc software (Epi Info 2005 v3.3.2; Centers of Disease Control and Prevention, Atlanta, GA, USA). Multivariable linear regression models were carried out for adjustment of glucose for potential confounders like age, gender, BMI, and tobacco. In order to assess the genetic risk factor for diabetes and obesity, logistic regression analyses were applied to estimate the OR for each polymorphism. Because the associations between the SNPs and the outcomes have been previously reported, it is unlikely to detect effects due to statistical fluctuations only. Therefore, correction by multiple comparisons was not applied. Associations were considered statistically significant at a nominal *P* value ≤0.05. Haplotype analysis was performed using PLINK and Haploview software. The *t*-test, Chi-square test, Fischer exact test, and multivariable regressions were performed using the statistical software Stata 12.0 (StataCorp LP, College Station, TX, USA).

## 3. Results

### 3.1. Clinical and Anthropometrical Measures

General characteristics of the study population are shown in Table 1. The mean of age, BMI, glucose, HbA1c, triglycerides, LDL-C, and systolic blood pressure were significantly higher in subjects with diabetes as compared with control group. On the other hand, the HDL-C levels were found lower in diabetic subjects (*P* < 0.001), which together show some of the metabolic abnormalities associated with T2DM. Taking into account that obesity frequency was found higher in the control group (*P* < 0.0001), it was considered as a potential confounder in multivariate analyses.

### 3.2. Genotype Frequency in Diabetic and Obese Subjects

The distribution of the Native American (NAM), the European (EUR), and the African (AFR) individual admixture proportions was comparable between the diabetic subjects and controls (NAM: *P* = 0.2727; EUR: *P* = 0.2579; and AFR: *P* = 0.1917). The analysis did not include the individual ancestry proportions, which were not available for all the subjects.

Table 2 shows genotype and allelic frequency of the polymorphisms studied. Hardy Weinberg equilibrium (HWE) test showed no deviation inside the population. There was no association with diabetes and genotype frequency found in any of the polymorphisms studied (*P* > 0.05). However, taking into account that the polymorphisms studied have been associated with obesity, the genotype frequencies were analyzed according to obesity (Table 3). Lower frequency of the TT genotype in obese people was observed (4.7% versus 7.7%) which provides a significant protection against obesity (OR: 0.54, *P* = 0.012). A marginal association, but not significant (*P* = 0.06), was found with rs2071749 polymorphism and obesity. Therefore, subanalyses were carried out according to genetic inheritance models and diabetes and obesity status, and stratification by gender.

When CC + CT genotype of the rs7211 polymorphism was compared with TT, lower frequency of TT genotype associated with lower BMI (TT: 27.6 ± 4 versus CC + CT: 29 ± 5, *P* = 0.011) and higher HDL-C levels (TT: 58 ± 13 ± 4 versus CC + CT: 49 ± 12, *P* = 0.022) in obese subjects without diabetes was found. The OR revealed that TT genotype is a protective factor against obesity in nondiabetic individuals (Table 4). In the multivariable analysis, genotype (as recessive model) was negatively associated with BMI, as well as HDL-C levels in nondiabetic subjects (Table 5). However, these associations were not found in diabetic or diabetic obese subjects.

After gender stratification, CC genotype was higher in obese women compared with nonobese women (Table 4). TT genotype was also lower in obese women (TT frequency in obese women = 5.12 versus nonobese women = 9.23, *P* = 0.028) but significance was achieved when multivariate analysis and logistic regression were carried out including genotype as dominant model. Results showed that carrying one copy of T allele decreases the risk of obesity in women (Table 4). However the association of BMI with genotype was weak and explains just 0.48% of BMI variability (Table 5).

The rs2071749 polymorphism in the HMOX1 gene was significantly associated with obesity when it was analyzed under a dominant model (Table 6). AA carriers showed lower BMI than AG + GG carriers and had lower risk of obesity. The association of the rs2071749 polymorphism persists with obesity, after being adjusted by age, gender, HDL-C levels, LDL-C levels, and triglycerides concentrations.

The rs6721961 polymorphism of the Nrf2 gene was associated with diabetes in men but not in women, after stratification by gender (Table 7). CC carriers had higher glucose levels in comparison with CA + CC carriers when genotype was compared as dominant model. Thus, the presence of the A allele represents a protective factor against diabetes in men. The pairwise linkage disequilibrium (LD) analysis of the rs6721961 and rs2364723 in NRF2 gene revealed a low LD between them (*r* ^2^ = 0.31). Additionally, the analyses of potential haplotype effects showed no association with diabetes (*P* = 0.74) or obesity (*P* = 0.52).

## 4. Discussion

Oxidative stress is one of the major metabolic factors that lead to the onset of chronic disease like insulin resistance, hypertension, metabolic syndrome, prediabetes, diabetes, and its complications. It has been recognized that the antioxidant system is abated in diabetes and obesity, accompanied by an increased production of inflammatory cytokines. A defect in Nrf2 activation in many organs has been documented widely in experimental models of insulin resistance and diabetes leading to decreased expression of its target genes [31–34]. In this study, it was found that the rs6721961 (−617C/A) polymorphism of Nrf2 gene was associated with diabetes in Mexican mestizo men, while the rs7211 polymorphism of TXNIP gene and the rs2071749 polymorphism of HMOX1 gene were associated with obesity. Nevertheless, the polymorphisms rs1800566 (NQO1) and rs2364723 (Nrf2) were not found to be associated with diabetes or obesity.

Wang et al. [35] did not found association between the rs1800566 polymorphism in NQO1 gene and the risk of T2DM in Chinese population. On the other hand, Kim [36] reported that there were no associations between the rs1800566 polymorphism and BMI, blood pressure, lipid profile, HbA1c, postprandial glucose, and homeostasis model assessment-insulin resistance (HOMA-IR). Nevertheless, Martínez-Hernández et al. [18] showed that the T allele of the rs1800566 polymorphism was associated with increased triglycerides levels and decreased HDL-C levels in Mexican mestizo individuals with metabolic syndrome. However, in this study this association was not found and we suggest that this difference is due to the fact that 57% and 47% of the subjects recruited in our study were under medical treatment with fibrates and statins, respectively, and attend to clinical detection annually.

The rs7211 has been poorly studied in diabetic and obese subjects. First, van Greevenbroek et al. [23] found that triglycerides levels were higher in diabetic subjects with the T allele than the C allele carriers, which was associated with higher glucose concentrations. Subsequently, Ferreira et al. [24] found that the rs7211 was associated in Brazilian subjects with diabetes and hypertension, and T carriers showed higher concentrations of blood glucose and systolic blood pressure. Das et al. [37] reported that, in European-American or African-American subjects recruited in USA, the TXNIP gene expression was negatively correlated with obesity. In this study, the T allele was found as a protection factor against metabolic traits like BMI and HDL-C. The T allele carriers showed that lower BMI values and HDL-C concentrations in nondiabetic subjects and in women are still associated with obesity after adjustment by confounding factors. Some authors reported that this polymorphism can increase TXNIP expression [22], but this still remains unclear. However, this is the first report in which it was found that T allele is a protective factor against obesity and we suggest that TXNIP expression or function would be affected allowing TXN to exert its antioxidant action.

The SNP rs2071746 (T-413A) and (GT)_*n*_ microsatellite are polymorphisms in the promoter region of HMOX1 gene which can modulate its transcriptional activity and have been associated with various human diseases [19]. Lin et al. [20] showed that the (GT)_*n*_ repeat in the HMOX1 promoter was significantly associated with essential hypertension, systolic blood pressure, and diastolic blood pressure, whereas the other two SNPs rs2071746 and rs2071749 were not associated. Although the rs2071749 has been poorly studied with metabolic traits, in this study it was found that AA carriers showed lower BMI values which were associated with the risk of presenting obesity, whereas GG carriers showed higher BMI values. This is the first report showing the association of the rs2071749 polymorphism and obesity. This polymorphism is an intronic tag SNP and it has reported that it is in LD with the rs3761439, which is located in the promoter region of the HMOX1 gene. The consensus sequence of a nuclear factor *κ*B (NF-*κ*B) binding site to HMOX1 is altered by rs3761439 polymorphism that increases the risk of developing impaired lung function [38]. This suggests that the mechanisms by which this polymorphism could be involved in obesity may be secondary to increased oxidative stress and inflammation. The results in this work open the gate to new investigations about the role of the HMOX1 gene in Mexican obese patients.

NRF2 gene regulates the enzymes studied here. Many SNPs have been identified in this gene. The rs35652124 and rs6721961 SNPs are predicted to affect Nrf2 myeloid zinc finger 1 (MZF1) and antioxidant response elements- (ARE-) like promoter binding sites, respectively. These SNPs affect the efficient binding of proteins such as Nrf2 to the MZF1 and ARE-like promoter binding sites [39].

In metabolic diseases, the rs6721961 polymorphism has been associated with blood pressure in Japanese subjects [39]. Wang et al. [7] showed that the rs6721961 polymorphism in the NRF2 gene was significantly associated with oxidative stress, antioxidant status, and risk of newly diagnosed T2DM, as well as with impaired insulin secretory capacity and increased insulin resistance in T2DM patients of a Chinese population. Individuals with the CC genotype had lower total antioxidant capacity, glutathione levels and superoxide dismutase, catalase, and glutathione peroxidase activities as well as lower homeostasis model assessment of *β*-cell function index (HOMA-*β*) in comparison with individuals with the CC genotype. Those with the AA genotype also had a higher malondialdehyde concentration and HOMA-IR index values. The frequency of allele A was significantly higher in T2DM subjects (29.4%). Individuals with the AA genotype had a significantly higher risk of developing T2DM, relative to those with the CC genotype, even after adjusting for known T2DM risk factors. However, this investigation showed that the AA carriers had lower glucose concentrations and the OR revealed that A carriers had lower risk of developing diabetes in men after stratification by gender. We had the limitation that Nrf2 gene expression was not determined in these patients and it is essential to elucidate the mechanisms by which the rs6721961 polymorphism can exert its protective effect in our population. Larger sample size could support this.

We know that this research has some limitations. The gene expressions and activities were not determined and just candidate genes were included. Dietary information was not available in all patients. However, this study opens the gate to new researches about the role of HMOX1, TXNIP, and Nrf2 in obesity, diabetes, and worse metabolic traits.

## 5. Conclusions

This study shows that the rs7211 polymorphism in the TXNIP gene and the rs2071749 of the HMOX1 gene are associated with obesity in Mexican mestizo people. In this sense, rs2071749 increases the risk of having obesity and the rs7211 polymorphism may be a protective factor to develop obesity and to have lower concentrations of HDL-C in Mexican mestizo women. In addition, rs6721961 gen polymorphism in Nrf2 was negatively associated with diabetes in men. All of these results open the doors for new research, taking into account the effect on metabolic traits, and may be useful tools to design new therapeutic strategies in obesity and diabetes type 2.

## Figures and Tables

**Table 1:** Clinical and anthropometric characteristics of the study groups.

Characteristic	Control	Diabetes	*P*
*n*	1020	627	
Age (years)	42.1 ± 7.3	53.2 ± 9.4	<0.001
Males, *n* (%)	474 (46)	302 (48)	0.759
BMI (kg·m^−2^)	28.7 ± 4.9	29.7 ± 6.9	0.0016
Obesity, *n* (%)	350 (34)	289 (46)	<0.001
Glucose (mg·dL^−1^)	95.5 ± 9.6	154 ± 73	<0.001
HbA1c	4.45 ± 0.8	7.27 ± 2.1	<0.001
Triglycerides (mg·dL^−1^)	183 ± 122	214 ± 140	<0.001
Total cholesterol (mg·dL^−1^)	201 ± 43	198 ± 52	0.2257
HDL-C (mg·dL^−1^)	49.5 ± 12.5	45.4 ± 13	<0.001
LDL-C (mg·dL^−1^)	129.2 ± 36	152.5 ± 56	<0.001
Creatinine (mg·dL^−1^)	1.17 ± 0.22	1.2 ± 0.92	0.4225
SBP	116 ± 14	121 ± 16	<0.001
DBP	77 ± 10	77.4 ± 10.6	0.102
Smoking, *n* (%)	256 (25)	159 (25.3)	0.918

Continuous variables are presented as means ± SD and categorical variables as numbers (percentage). BMI, body mass index; HbA1c, glycated hemoglobin; HDL-C, high-density lipoprotein cholesterol; LDL-C, low-density lipoprotein cholesterol; SBP, systolic blood pressure; DBP: diastolic blood pressure.

**Table 2:** Genotype frequencies of the polymorphisms studied in diabetic patients and controls.

Gene/polymorphism	Genotypes alleles	Diabetes	Controls	OR (95% CI)	*P*	*P* of HWE
TXNIP rs7211	CC, *n* (%)	345 (55.4)	528 (54.5)	Reference		0.880
	CT, *n* (%)	239 (38.3)	376 (38.8)	0.88 (0.6–1.3)	0.577	
	TT, *n* (%)	39 (6.3)	65 (6.7)	0.9 (0.8–1.2)	0.776	

NQO1 rs1800566	CC, *n* (%)	216 (34.7)	327 (33)	Reference		0.406
	CT, *n* (%)	288 (46.2)	483 (48.6)	0.90 (0.7–1.1)	0.373	
	TT, *n* (%)	119 (19.1)	183 (18.4)	0.98 (0.7–1.3)	0.915	

HMOX1 rs2071749	AA, *n* (%)	269 (43.8)	413 (43.2)	Reference		0.625
	AG, *n* (%)	267 (43.5)	417 (43.6)	1.05 (0.76–1.4)	0.757	
	GG, *n* (%)	78 (12.7)	126 (13.2)	1.03 (0.74–1.4)	0.837	

NRF2 rs2364723	CC, *n* (%)	210 (33.6)	301 (30.3)	Reference		0.092
	CG, *n* (%)	286 (56.8)	471 (47.5)	0.87 (0.7–1.1)	0.236	
	GG, *n* (%)	129 (20.6)	220 (22.2)	0.84 (0.63–1.1)	0.223	

NRF2 rs6721961	CC, *n* (%)	407 (65.3)	618 (62.5)	Reference		0.281
	CA, *n* (%)	189 (30.4)	317 (32)	0.9 (0.7–1.1)	0.374	
	AA, *n* (%)	27 (4.3)	54 (5.5)	0.76 (0.5–1.2)	0.259	

CI, confidence interval; HWE, Hardy-Weinberg equilibrium; HMOX1, heme oxygenase-1; NQO1, NAD(P)H quinone oxidoreductase 1; NRF2, Nuclear factor-erythroid 2- (NF-E2-) related factor 2; OR, odds ratio; and TXNIP, thioredoxin interacting protein.

**Table 3:** Genotype and allele frequencies of the polymorphisms studied in obese and nonobese subjects.

Gene/polymorphism	Genotype	Obesity	No obesity	OR (95% CI)	*P*
TRXNIP rs7211	CC, *n* (%)	350 (56.6)	523 (53.7)	Reference	
	CT, *n* (%)	239 (38.7)	376 (38.6)	0.95 (0.7–1.2)	0.627
	TT, *n* (%)	29 (4.7)	75 (7.7)	0.56 (0.35–0.87)	0.012

NQO1 rs1800566	CC, *n* (%)	212 (34.2)	331 (33.3)	Reference	
	CT, *n* (%)	302 (48.8)	469 (47)	1 (0.8–1.25)	0.963
	TT, *n* (%)	106 (17)	196 (19.7)	0.84 (0.6–1.1)	0.257

HMOX1 rs2071749	AA, *n* (%)	261 (40)	419 (45.7)	Reference	
	AG, *n* (%)	306 (46.9)	378 (41.3)	1.3 (1–1.6)	0.019
	GG, *n* (%)	85 (13.1)	119 (13)	1.1 (0.8–1.5)	1.14

NRF2 rs2364723	CC, *n* (%)	194 (31)	317 (32)	Reference	
	CG, *n* (%)	300 (48)	457 (46)	1.1 (0.85–1.35)	0.551
	GG, *n* (%)	131 (21)	210 (22)	0.98 (0.7–1.3)	0.899

NRF2 rs6721961	CC, *n* (%)	390 (63.1)	635 (63.9)	Reference	
	CA, *n* (%)	195 (31.6)	311 (31.3)	1 (0.8–1.3)	0.853
	AA, *n* (%)	33 (5.3)	48 (4.8)	1.1 (0.7–1.7)	0.631

CI, confidence interval; HMOX1, heme oxygenase-1; NQO1, NAD(P)H quinone oxidoreductase 1; NRF2, Nuclear factor-erythroid 2- (NF-E2-) related factor 2; OR, odds ratio; and TXNIP, thioredoxin interacting protein.

**Table 4:** Genotype frequency of the rs7211 polymorphism in subjects without diabetes and women.

	Obese	Nonobese	Crude OR (95% CI)	*P*	Adjusted^a^ OR (95% CI)	*P*
Nondiabetic						
CC	189 (56.6)	339 (53.4)	Reference		Reference	
CT	133 (40)	241 (38)	0.98 (0.74–1.3)	0.903	1 (0.76–1.4)	0.863
TT	55 (8.6)	12 (3.6)	0.3 (0.2–0.7)	0.004	0.3 (0.15–0.7)	0.003
CC + CT	322 (96.4)	580 (91.5)	0.4 (0.2–0.76)	0.005	0.3 (0.15–0.7)	0.003

Women						
CC	197 (59)	259 (51)	Reference		Reference	
CT	118 (36)	203 (40)	0.7 (0.6–1)	0.072	0.9 (0.6–1.2)	0.418
TT	17 (5)	47 (9)	0.5 (0.26–0.85)	0.013	0.5 (0.25–0.96)	0.04
CT + TT	135 (41)	250 (49)	0.70 (0.5–0.9)	0.016	0.7 (0.5–0.96)	0.028

CI, confidence interval; OR, odds ratio.

^a^Obesity in logistic regression was adjusted by age, gender (except in women model), glucose, triglycerides, LDL-C, and HDL-C levels.

**Table 5:** Multiple linear regression of BMI as dependent variable in nondiabetic subjects and women.

	*β*	*P*	*R* ^2^
Nondiabetic subjects			
HDL-C	−0.18	<0.001	0.11
Glucose	0.248	<0.001	
rs7211 (CT + TT: 0, TT: 1)	−0.07	0.033	

Women			
HDL	−0.12	0.003	0.048
Glucose	0.13	0.001	
rs7211 (CC: 0, CT + TT: 1)	−0.08	0.037	

Models also include age, gender (in nondiabetic subjects model), LDL-C, and triglycerides. Significant variables were presented. BMI, body mass index; HDL-C, high-density lipoprotein cholesterol.

**Table 6:** Association of the rs2071749 polymorphism of the HMOX1 gene with obesity.

Variable	Obese, *n* (%)	Nonobese, *n* (%)	*P*	BMI	*P*
AA	261 (40)	410 (46)	0.026	28.9 ± 4.9	0.027
AG + GG	390 (60)	497 (54)		29.5 ± 5.3	

Crude OR (95% CI)	1.25 (1.02–1.54)		0.026		
OR adjusted^a^ (95% CI)	1.34 (1.06–1.7)		0.013		
Beta coefficient/*R* ^2,b^	0.063/0.03		0.023		

BMI values are presented as mean ± SD. BMI, body mass index, CI: confidence interval, and OR: odds ratio.

^a^Obesity in logistic regression was adjusted by age, gender, glucose, triglycerides, and LDL-C and HDL-C levels. Genotype was included as dominant model in logistic and multivariate analysis.

^b^Linear regression of BMI as dependent variable was adjusted by age, gender, glucose, triglycerides, and LDL-C and HDL-C levels.

**Table 7:** Association of the rs6721961 polymorphism of the Nrf2 gene with diabetes in men.

Variable	Diabetic, *n* (%)	Nondiabetic, *n* (%)	*P*	Glucose	*P*
CC	203 (69)	271 (58)	0.011	126.6 ± 59	0.056^b^
CA	79 (27)	171 (37)		116 ± 56	
AA	12 (4)	23 (5)		105 ± 32	
CA + AA	91 (31)	194 (42)	0.003	114.4 ± 53	0.031^c^

Crude OR (95% CI)	0.62 (0.46–0.85)		0.003		
OR adjusted^a^ (95% CI)	0.56 (0.38–0.82)		0.003		
Crude beta coefficient	−0.078		0.031		

Glucose values (mg·dL^−1^) are presented as mean ± SD. CI: confidence interval; OR: odds ratio.

^a^Diabetes in logistic regression was adjusted by age, triglycerides, and LDL-C and HDL-C levels. Genotype was included as dominant model in logistic and linear regression.

^b^ *P* value when comparing each genotype.

^c^ *P* value in comparison of CC with CA + AA.
