# Corrigendum to “Association of Nuclear Factor-Erythroid 2-Related Factor 2, Thioredoxin Interacting Protein, and Heme Oxygenase-1 Gene Polymorphisms with Diabetes and Obesity in Mexican Patients”

**DOI:** 10.1155/2017/7543194

**Published:** 2017-05-30

**Authors:** Angélica Saraí Jiménez-Osorio, Susana González-Reyes, Wylly Ramsés García-Niño, Hortensia Moreno-Macías, Martha Eunice Rodríguez-Arellano, Gilberto Vargas-Alarcón, Joaquín Zúñiga, Rodrigo Barquera, José Pedraza-Chaverri, Juan Pablo Meza-Espinoza, Evelia Leal-Ugarte, Valeria Peralta-Leal

**Affiliations:** ^1^Department of Biology, National Autonomous University of Mexico (UNAM), 04510 Mexico City, DF, Mexico; ^2^Economy Department, Autonomous Metropolitan University-Iztapalapa, 09340 Mexico City, DF, Mexico; ^3^Research Department, Regional Hospital “Lic. Adolfo López Mateos”, ISSSTE, 01030 Mexico City, DF, Mexico; ^4^Molecular Biology Department, National Institute of Cardiology “Ignacio Chávez”, 14080 Mexico City, DF, Mexico; ^5^Department of Immunology, National Institute of Respiratory Diseases “Ismael Cosío Villegas”, 14080 Mexico City, DF, Mexico; ^6^Molecular Genetics Laboratory, National School of Anthropology and History, 14030 Mexico City, DF, Mexico; ^7^Departamento de Genética, Facultad de Medicina e Ingeniería en Sistemas Computacionales de Matamoros, Universidad Autónoma de Tamaulipas, Sendero Nacional km 3, 87349 Matamoros, TAMPS, Mexico

In the article titled “Association of Nuclear Factor-Erythroid 2-Related Factor 2, Thioredoxin Interacting Protein, and Heme Oxygenase-1 Gene Polymorphisms with Diabetes and Obesity in Mexican Patients,” [1] typing errors were found after subsequent analyses of the entire dataset. These issues were raised to the attention of the original authors by Drs. Meza-Espinoza, Leal-Ugarte, and Peralta-Leal of the Universidad Autónoma de Tamaulipas. The allelic frequencies were added and the corrected data are shown in italics in Tables 2, 3, and 4.

It is important to remark that the model (recessive for allele T, Table 4) was calculated taking in count that CC + CT = 0. When CC is considered as the risk allele the OR increases to 2.4 (CI: 1.28–4.64, *P* = 0.006) under a dominant model (CC + CT = 1 versus TT = 0). This reveals that the TT genotype in this study shows a protective factor against obesity and the genotype CC could be considered as the risk factor for obesity, as was stated in the original article.

The last paragraph of Section 3 should be “CC carriers had higher glucose levels in comparison with CA + *AA* carriers when genotype was compared as dominant model.”

Finally, in Section 4 (Discussion) when Wang et al. [7] is cited, the correct statement is the following:

“Individuals with CC genotype had lower total antioxidant capacity, glutathione levels, superoxide dismutase, catalase, and glutathione peroxidase activities as well as lower homeostasis model assessment of *β*-cell function index (HOMA-*β*) in comparison with individuals with the AA genotype.”

## Figures and Tables

**Table 2:** Genotype and allele frequencies of the polymorphisms studied in diabetic patients and controls.

Gene/polymorphism	Genotypes	Diabetes	Controls	OR (95% CI)	*P*	*P* of HWE
	Alleles	*n* (%)	*n* (%)			
TXNIP rs7211	CC	345 (55.4)	528 (54.5)	Reference		0.880
	CT	239 (38.4)	376 (38.8)	*0.97 (0.78–1.2)*	*0.798*	
	TT	39 (6.2)	65 (6.7)	*0.92 (0.60–1.39)*	*0.690*	
	*C*	*939 (74.6)*	*1432 (73.9)*	*Reference*		
	*T*	*317 (25.4)*	*506 (26.1)*	*0.966 (1.82–1.37)*	*0.674*	

NQO1 rs1800566	CC	216 (34.7)	327 (33)	Reference		0.406
	CT	288 (46.2)	483 (48.6)	0.90 (0.7–1.1)	0.373	
	TT	119 (19.1)	183 (18.4)	0.98 (0.7–1.3)	0.915	
	*C*	*720 (57.8)*	*1137 (57.25)*	*Reference*		
	*T*	*526 (42.2)*	*849 (42.75)*	*0.98 (0.85–1.13)*	*0.765*	

HMOX-1 rs2071749	AA	269 (43.8)	413 (43.2)	Reference		0.625
	AG	267 (43.5)	417 (43.6)	*0.98 (0.79–1.2)*	*0.877*	
	GG	78 (12.7)	126 (13.2)	*0.95 (0.69–1.3)*	*0.757*	
	*A*	*805 (65.5)*	*1243 (65)*	*Reference*		
	*G*	*423 (34.5)*	*669 (35)*	*0.97 (0.84–1.13)*	*0.755*	

NRF2 rs2364723	CC	210 (33.6)	301 (30.3)	Reference		0.092
	CG	286 (*45.7*)	471 (47.5)	0.87 (0.7–1.1)	0.236	
	GG	129 (20.6)	220 (22.2)	0.84 (0.63–1.1)	0.223	
	*C*	*706 (56.5)*	*1073 (54)*	*Reference*		
	*G*	*544 (43.5)*	*911 (46)*	*0.91 (0.79–1.05)*	*0.182*	

NRF2 rs6721961	CC	407 (65.3)	618 (62.5)	Reference		0.281
	CA	189 (30.4)	317 (32)	0.90 (0.7–1.1)	0.374	
	AA	27 (4.3)	54 (5.5)	0.76 (0.5–1.2)	0.259	
	*C*	*1003 (80.5)*	*1553 (78.5)*	*Reference*		
	*A*	*243 (19.5)*	*425 (21.5)*	*0.88 (0.74–1.05)*	*0.176*	

CI, confidence interval; HWE, Hardy-Weinberg equilibrium; HMOX-1, heme oxygenase 1; NQO1, NAD(P)H quinone oxidoreductase 1; NRF2, nuclear factor-erythroid 2- (NF-E2-) related factor 2; OR, odds ratio; and TXNIP, thioredoxin-interacting protein.

**Table 3:** Genotype and allele frequencies of the polymorphisms studied in obese and nonobese subjects.

Gene/polymorphism	Genotype	Obesity	No obesity	OR (95% CI)	*P*
	Alleles	*n* (%)	*n* (%)		
TRXNIP rs7211	CC	350 (56.6)	523 (53.7)	Reference	
	CT	239 (38.7)	376 (38.6)	*0.95 (0.77–1.17)*	*0.633*
	TT	29 (4.7)	75 (7.7)	*0.57 (0.37–0.9)*	*0.017*
	*C*	*239 (76)*	*1422 (73)*	*Reference*	
	*T*	*297 (24)*	*526 (27)*	*0.85 (0.72–1)*	*0.061*

NQO1 rs1800566	CC	212 (34.2)	331 (33.3)	Reference	
	CT	302 (48.8)	469 (47)	1 (0.8–1.25)	0.963
	TT	106 (17)	196 (19.7)	0.84 (0.6–1.1)	0.257
	*C*	*726 (58.5)*	*1131 (56.8)*	*Reference*	
	*T*	*514 (41.5)*	*561 (43.2)*	*0.93 (0.8–1.07)*	*0.322*

HMOX-1 rs2071749	AA	261 (40)	419 (45.7)	Reference	
	AG	305 (46.9)	378 (41.3)	1.3 (1–1.6)	0.019
	GG	85 (13.1)	119 (13)	1.1 (0.8–1.5)	*0.399*
	*G*	*827 (63.5)*	*1216 (66.4)*	*Reference*	
	*A*	*475 (36.5)*	*616 (36.6)*	*1.13 (0.97–1.31)*	*0.097*

NRF2 rs2364723	CC	194 (31)	317 (32)	Reference	
	CG	300 (48)	457 (46)	1.1 (0.85–1.35)	0.551
	GG	131 (21)	*218* (22)	0.98 (0.7–1.3)	0.899
	*C*	*688 (55)*	*1091 (55)*	*Reference*	
	*G*	*562 (45)*	*893 (45)*	*0.99 (0.86–1.15)*	*0.699*

NRF2 rs6721961	CC	390 (63.1)	635 (63.9)	Reference	
	CA	195 (31.6)	311 (31.3)	1 (0.8–1.3)	0.853
	AA	33 (5.3)	48 (4.8)	1.1 (0.7–1.7)	0.631
	*C*	*975 (78.9)*	*1581 (79.5)*	*Reference*	
	*A*	*261 (21.1)*	*407 (20.5)*	*1.04 (0.87–1.23)*	*0.661*

TXNIP, thioredoxin-interacting protein; NQO1, NAD(P)H quinone oxidoreductase 1; HMOX-1, heme oxygenase 1; NRF2, nuclear factor-erythroid 2- (NF-E2-) related factor 2.

**Table 4:** Genotype frequency of the rs7211 polymorphism in subjects without diabetes and women.

	Obese	Nonobese	Crude OR (95% CI)	*P*	Adjusted^a^ OR (95% CI)	*P*
Nondiabetic						
CC	189 (56.6)	339 (53.4)	Reference		Reference	
CT	133 *(39.8)*	*243 (38.3)*	*0.98 (0.75–1.3)*	*0.896*	1 (0.77–1.4)	0.863
TT	*12 (3.6)*	*53 (8.3)*	*0.4 (0.21–0.77)*	*0.007*	0.3 (0.15–0.7)	0.003
CC + CT = 0 versus TT = 1	*322 (96.4)*	*582 (91.5)*	*0.4 (0.21–0.76)*	*0.006*	*0.39 (0.18–0.8)*	*0.014*
Women						
CC	197 (59)	259 (51)	Reference		Reference	
CT	118 (36)	203 (40)	0.7 (0.6–1)	0.072	0.9 (0.6–1.2)	0.418
TT	17 (5)	47 (9)	0.5 (0.26–0.85)	0.013	0.5 (0.25–0.96)	0.04
CT + TT	135 (41)	250 (49)	0.70 (0.5–0.9)	0.016	0.7 (0.5–0.96)	0.028

CI, confidence interval; OR, odds ratio.

^a^Obesity in logistic regression was adjusted by age, gender (except in women model), glucose, triglycerides, LDL-C, and HDL-C levels.
